# Human melanopsin (OPN4) gene polymorphisms: a systematic review

**DOI:** 10.3389/fnins.2025.1581266

**Published:** 2025-06-10

**Authors:** Kevin R. Lucio-Enríquez, Mariazel Rubio-Valles, Arnulfo Ramos-Jiménez, Jorge A. Pérez-León

**Affiliations:** ^1^Chemical Biological Sciences PhD Graduate Program, Department of Chemical Sciences, Biomedical Sciences Institute, Ciudad Juarez Autonomous University, Chihuahua, Mexico; ^2^Physical Activity Sciences for Health PhD Graduate Program, Faculty of Physical Culture Sciences, Chihuahua Autonomous University, Chihuahua, Mexico; ^3^Department of Health Sciences, Biomedical Sciences Institute, Ciudad Juarez Autonomous University, Chihuahua, Mexico; ^4^Department of Chemical Sciences, Biomedical Sciences Institute, Ciudad Juarez Autonomous University, Chihuahua, Mexico

**Keywords:** melanopsin, polymorphisms, non-visual function, human, circadian rhythms

## Abstract

The melanopsin (OPN4) gene is crucial in visual and non-visual processes. Certain single-nucleotide polymorphisms (SNPs) of this gene have been linked to altered light sensitivity, photoentrainment, sleep disorders, and metabolic problems, which suggests a systemic effect of light exposure. The aim of this systematic review is to explore the current literature regarding the OPN4 gene and its SNPs, along with their associations with health-related problems. The literature search was conducted in PubMed and ScienceDirect databases using the following key terms: (“Melanopsin” OR “OPN4” OR “Opsin 4”) AND (“Polymorphism” OR “SNP” OR “Variant”). The publications were from January 1998 to February 2025. We identified 763 studies, and after screening titles, abstracts, full texts, and the inclusion and exclusion criteria, nine studies were included in the review. The review was conducted by two independent reviewers following the PRISMA guidelines. Our review revealed that some SNPs of the OPN4 gene, such as P10L, I394T, and R168C, are associated with affective states, changes in chronotype, and sleep disorders: P10L variant has been associated to seasonal affective disorder (SAD), chronotype, and chronic insomnia; I394T variant has been linked to the pupillary light response (PLR) and sleep/wake timing, while R168C variant has been associated with delayed sleep-wake phase disorder (DSWPD). Currently, the remaining SNPs have no reported associations, and the existing literature does not describe any specific molecular mechanisms through which these variants could modulate or alter OPN4 function. Future research should aim to explore these identified SNPs with alternative associations related to OPN4 functions.

## Introduction

**Figure 1:**
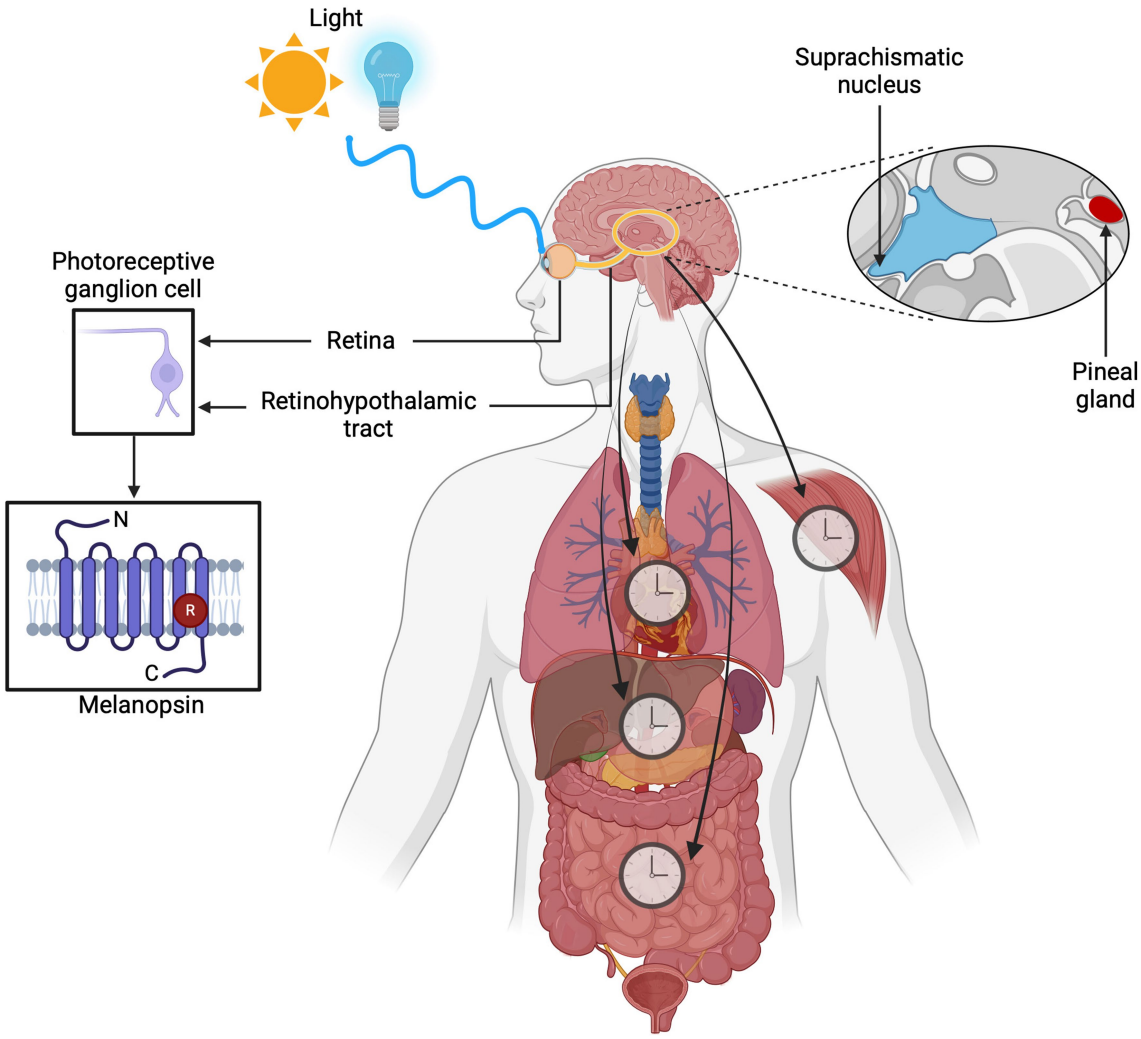
Overview of the role of melanopsin (OPN4) in circadian photoentrainment. Intrinsically photosensitive retinal ganglion cells (ipRGCs) containing melanopsin detect light at ~480 nm through the retina and transmit signals via the retinohypothalamic tract (RHT) to the suprachiasmatic nucleus (SCN). This process regulates key physiological processes such as sleep-wake cycles, hormone secretion, eating behavior, weight management, cognitive performance, and emotional health.

Light regulates several physiological processes, many of which can persist even in the absence of vision. Opsins, which are membrane photosensitive proteins, can help with this task. Because they are G protein-coupled receptors (GPCRs), after absorbing photons, they generate a cellular response and a signaling cascade known as phototransduction (Haltaufderhyde et al., 2015; Moraes et al., 2021). Some opsins mediate visual functions, while others are involved in non-visual processes (Karthikeyan et al., 2023). Melanopsin (OPN4) plays a crucial role in these (Spitschan, 2019). It was first discovered and identified in embryonic melanophores of *Xenopus laevis* (Provencio et al., 1998), and its recognized role as a non-visual photoreceptor increased. The mammalian eye detects light through three types of photoreceptors: cones, rods, and OPN4, containing ganglion cells, so-called intrinsically photosensitive retinal ganglion cells (ipRGCs). Furthermore, OPN4 has been found in all vertebrate groups (Bellingham et al., 2006). It differs from the visual receptors in the retina and is especially sensitive to wavelengths of blue light (Buhr, 2023). This sensitivity allows the PRGs to send information through the retinohypothalamic tract (RHT) directly to the suprachiasmatic nucleus (SCN). Thus, ipRGCs play an essential role in photoentrainment by influencing physiological regulation processes such as sleep, hormonal regulation, eating patterns, along with weight control and even cognitive functions and emotions (Figure 1) (Foster et al., 2020). In recent years, OPN4 has been found in extraocular regions (Leung and Montell, 2017), such as the brain (Nissilä et al., 2017), skin (Kusumoto et al., 2020), and adipose tissue (Ondrusova et al., 2017). This suggests that the light in the body can induce a physiological response through mechanisms mediated by extraocular photoreceptors.

The OPN4 gene in humans exhibits a variety of single-nucleotide polymorphisms (SNPs) (Rodgers et al., 2018a, 2018b), which are a type of mutation more frequently found in the population (>1%) (Karki et al., 2015). A mutation in this gene can affect the functional response to light (Smieszek et al., 2022). This review aims to gather all the information available on the OPN4 gene SNPs and their associations with light perception disorder.

## Methodology. Identification of manuscripts

The search was done using PubMed and ScienceDirect databases with the following key terms combination: (“Melanopsin” OR “OPN4” OR “Opsin 4”) AND (“Polymorphism” OR “SNP” OR “Variant”). The publication was delimited from January 1998 to February 2025, and two independent reviewers screened all titles and abstracts following the PRISMA guidelines (Page et al., 2021).

### Inclusion criteria

- Peer-reviewed articles.
- Clinical trials (randomized or non-randomized) or case reports.
- Studies involving human subjects.
- Studies that include multiple species, along with humans.
- Studies that include *in vivo*, *in vitro*, or *in silico*.

### Exclusion criteria

- Review articles.
- Documents written in languages other than English.

### Data extracted

The authors, year, SNPs, sample size, population, country, findings, or associations were noted. The result section recorded the SNPs and associations as outcomes. The information from the included articles was listed by publication date to provide chronological order (Table 1). The missing information was classified as: not reported (n.r.) and entered in the table to complete the sections.

**Table 1:** Overview of the included studies.

Study	SNPs	Participants/Country	Findings/Associations
Roecklein et al. (2009)	P10L (rs2675703)	*n* = 120/United States	Seasonal affective disorder (SAD)
Lee et al. (2013)	I394T (rs1079610)	*n* = 75/Japan	Pupillary light reflex
Higuchi et al. (2013)	I394T (rs1079610)	*n* = 73/Japan	Pupillary light response
Roecklein et al. (2013)	I394T (rs1079610) and P10L (rs2675703)	*n* = 30/United States	Seasonal affective disorder and pupillary light response
Lee et al. (2014)	I394T (rs1079610)	*n* = 348/Japan	Sleep/wake timing
Rodgers et al. (2018a)	P10L (rs2675703) and I394T (rs1079610)	*In vitro*	Light responses and NIF behaviors *in vivo*
Rodgers et al. (2018b)	P10L (rs2675703), L365V, I394T (rs1079610), R406W and D444G (rs12262894)	*In vitro*	Light perception
Gutiérrez-Amavizca et al. (2021)	P10L (rs2675703)	*n* = 127/Mexico	Chronic insomnia
Smieszek et al. (2022)	R168C (rs143641898)	*n* = 1/Not reported	Delayed sleep-wake phase disorder (DSWPD)

### Quality assessment

The study critically assessed information retrieval and methodological problems. We used the risk of bias (RoB) (McGuinness and Higgins, 2021) as a quality assessment template. The RoB tool consists of questions on seven domains of potential biases. Each question had to be answered with an assignment of either “Low,” “High,” or “Unclear.”

## Results

### Summary of included studies

**Figure 2:**
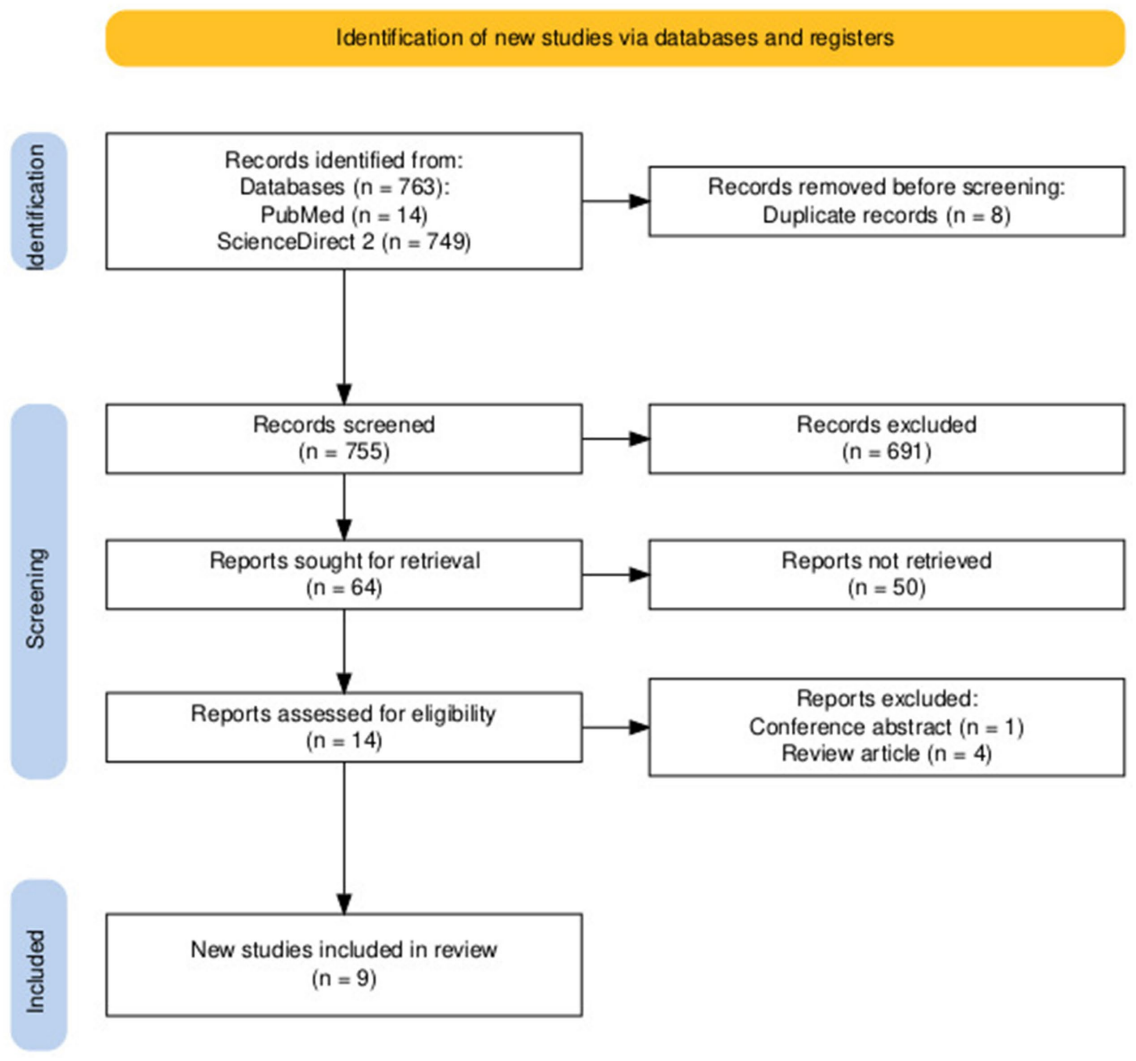
PRISMA flowchart of the literature research process.

Figure 2 summarizes the selection process of included studies. The initial search strategy yielded 763 studies. Duplicates were searched manually and subsequently excluded, 8 duplicates. After duplicates were removed, 755 studies remained. A total of 741 studies were excluded in this step based on screening of titles (691) and abstracts (50). Two independent researchers (KRLE and JAPL) screened the remaining studies; 14 studies were assessed for eligibility, and 5 studies were excluded (4 review articles and 1 conference abstract). A total of 9 studies were included in the systematic review.

### Detailed description of included and analyzed studies

Table 1 describes the analyzed studies. There is a predominance of the SNPs P10L and I394T studies, where each appeared in 5 and 6 articles, respectively, out of the 9 included studies. The number of participants ranges from 1 individual in a case study (Smieszek et al., 2022) to 348 subjects (Lee et al., 2014). Meanwhile, Rodgers et al. (2018a, 2018b) assessed the SNPs *in vitro*.

### Summary of risk of bias

**Figure 3:**
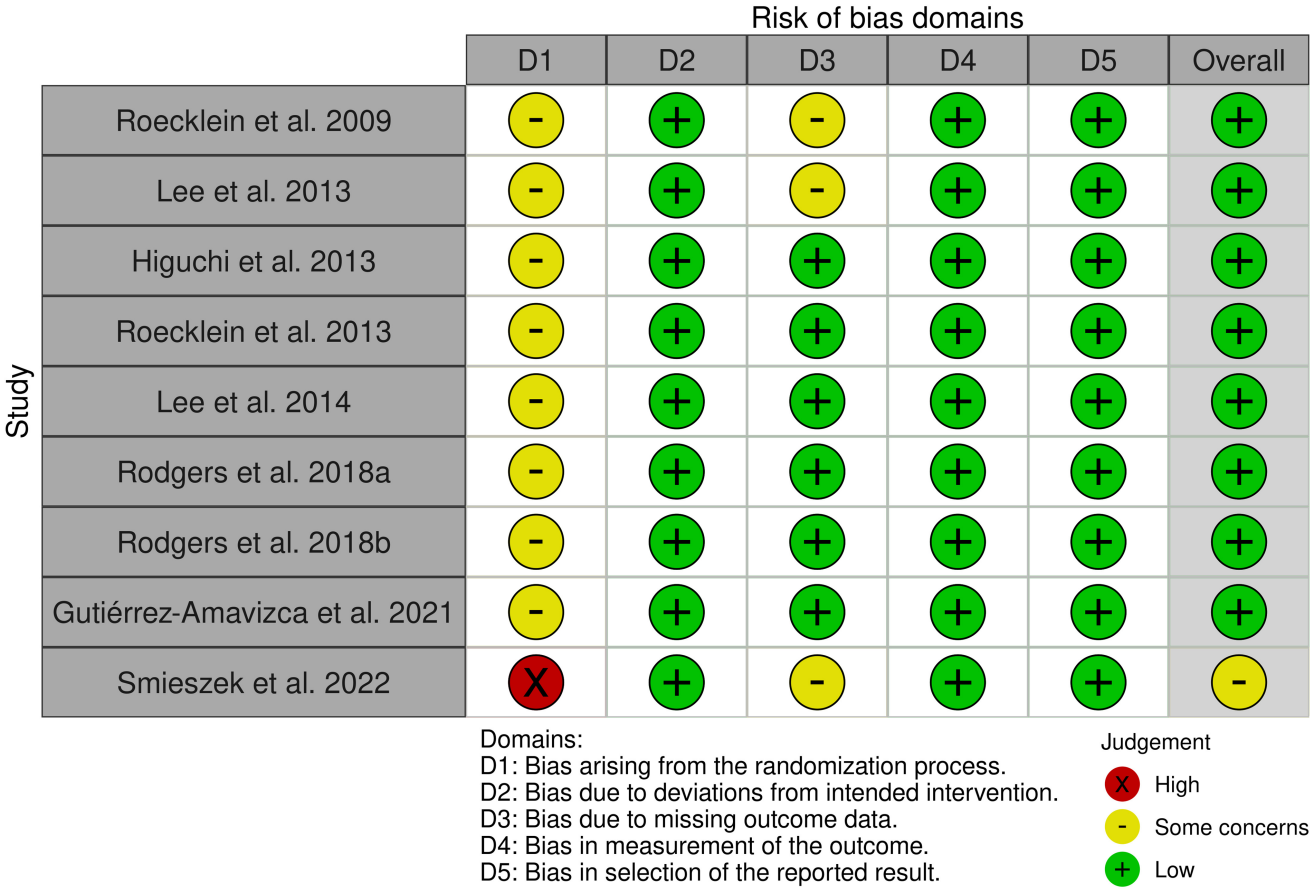
Traffic-light plot to risk of bias tool visualizes the assessment for each RoB question.

The RoB traffic-light plot (Figure 3) visualizes the assessment for each RoB question. There is a range of 13 years between the first and last study included in this review. All the studies had the disadvantage of their randomization process criteria due to the researchers’ need to control the subjects’ characteristics so that they could conduct their experiments. Even with those concerns in the process, neither the methodology nor the result seemed compromised, having a low risk of bias. Aside from that, their protocols were assessed as adequate.

### SNP P10L

**Figure 4:**
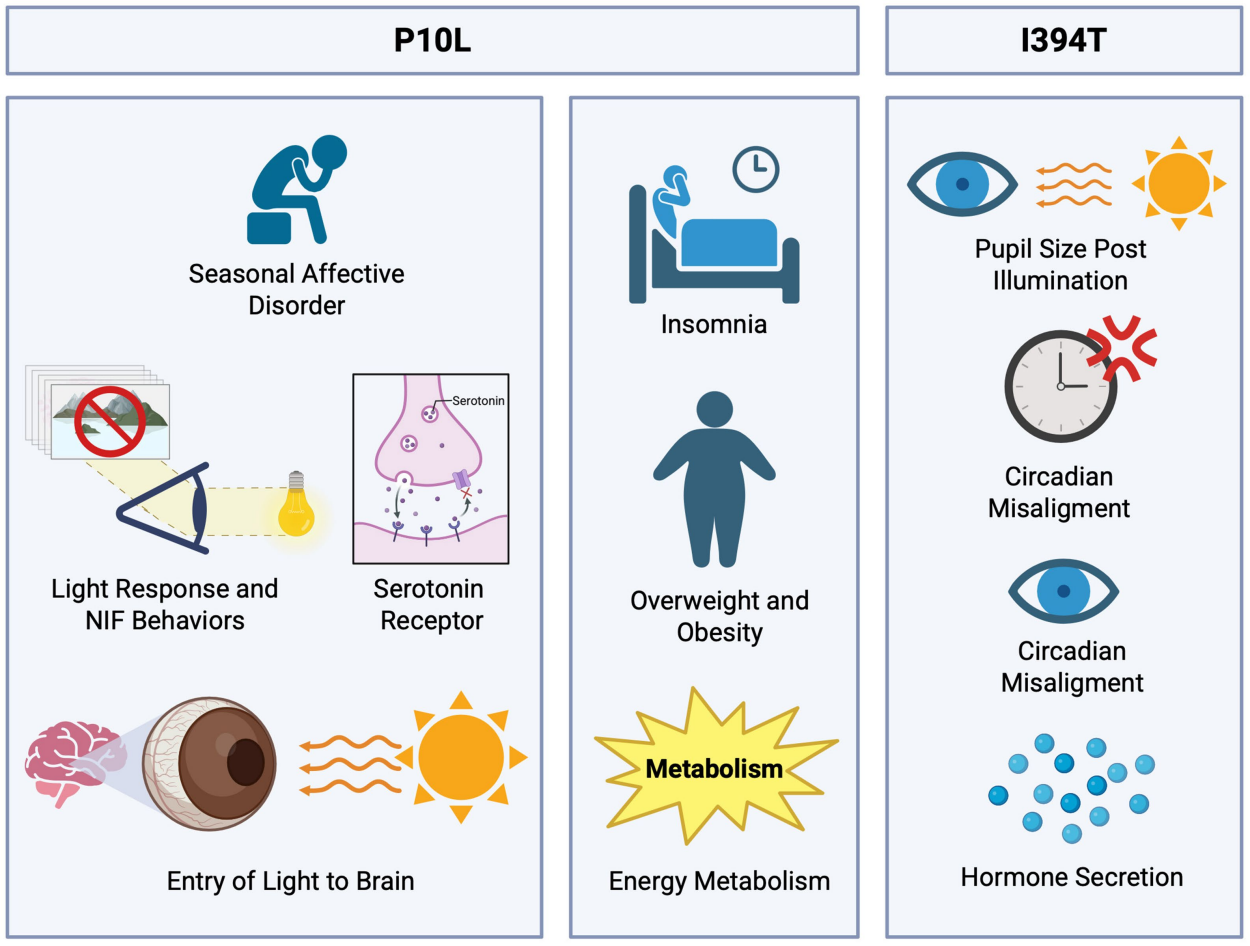
Functional roles of P10L and I394T polymorphisms in light-regulated physiological processes. The P10L polymorphism has been linked to seasonal affective disorder (SAD); this association highlights the role of genes involved in serotonin transport, serotonin receptors, and circadian clock regulation due to the decreased light input. Additionally, the P10L SNP shows a potential link to metabolic complications, as individuals with insomnia reported obesity or overweight. The I394T polymorphism has been associated with a reduction in pupil size and diminished light sensitivity, impacting the processes of circadian synchronization and hormone secretion.

The P10L SNP was first identified (Table 1) by Roecklein et al. (2009) as being associated with seasonal affective disorder (SAD). This is characterized by frequent depressive episodes, especially during fall and winter seasons (Gagné et al., 2011), showing a sudden remission during spring and summer. It has been speculated that light plays an important role in treating this disorder, making photoreceptors crucial because they process environmental light. It was considered that genes involved in this disorder were those of serotonin transporters, serotonin receptors, and clock genes (Figure 4). Thus, researchers hypothesized that ipRGCs are fundamental photoreceptors for non-visual functions and project light information directly to the SCN, and thus variations in the gene sequence could imply some modification in the function of circadian entrainment and light perception modulated by the pupillary light reflex, affecting the entry of light into the brain and increasing susceptibility to SAD. This could contribute to a higher light requirement for individuals to perform normal functions during low-light seasons. The study results showed that individuals with the P10L SNP, specifically with the T/T genotype, had a 5.6 times higher risk of developing SAD compared to the control group. It was also suggested that more distant regulatory regions of the gene might contribute to the hereditary risk of SAD associated with OPN4.

Subsequently, in the study of Gutiérrez-Amavizca et al. (2021), an association of P10L with chronic insomnia was determined in a Mexican population of 127 subjects through a combination of genotyping and questionnaires. According to Bidaki et al. (2012), insomnia is described as the difficulty in initiating or maintaining sleep, leading to poor quality and non-restorative sleep. Moreover, this disorder is quite prevalent, affecting up to 50% of the general population. This information, along with the previous association of the polymorphism (Roecklein et al., 2009) and the functions of OPN4, indicates a connection with the OPN4 gene, its SNPs, and insomnia. The results showed a strong association between the SNP P10L and insomnia, with a higher risk of insomnia in individuals with the T allele present. Additionally, there was a tendency in the insomnia group to be overweight or obese, with 44.8% of the insomnia group being overweight and 20.7% being obese, suggesting a possible implication in metabolic complications.

### SNP I394T

Another notable polymorphism is the I394T (rs1079610), substituting isoleucine with threonine at position 394. Studies have linked this SNP to the pupillary light reflex (PLR). Lee et al. (2013) investigated this association under diverse light conditions. The study was conducted with 195 participants exposed to different light intensities and wavelengths. The result indicated a significant reduction in the pupil size of individuals with TC + CC genotypes compared to those with the TT genotype (Figure 4).

Similarly, Higuchi et al. (2013) investigated the impact of this SNP on pupillary light response, finding various responses to the stimulation depending on light intensity. And while in this study the polymorphism in humans has been shown to affect PLP under low and high illuminance conditions, there are concerns regarding previous studies where melanopsin knockout (Opn4−/−) mice exhibit PLR deficits only under bright light (Lucas et al., 2003). The discrepancy may be due to its mechanism. While the precise mechanism by which the light perception is altered by the human I394T variant remains unknown, it is known that there is a partial loss of melanopsin function by disrupting ipRGCs without abolishment, only altering the light modulation levels. In contrast, the knockout mice completely lack melanopsin, leaving rods to entirely compensate during dim light conditions.

Roecklein et al. (2013) examined the post-illumination pupillary response (PIPR) in the population of individuals with seasonal affective disorder (SAD). This research was done in a smaller group of 30 individuals divided into 2 groups: subjects with SAD and a control group. The results indicated that the SAD group exhibited a lower PIPR response to blue light compared to the control group, and the response differed with the I394T genotype. This suggests that individuals with this condition have diminished light sensitivity, which influences the behavior and physiological response, affecting the mechanisms of circadian entrainment and hormone secretion (Figure 4) (Gnocchi and Bruscalupi, 2017).

### Photoentrainment and circadian rhythms

Circadian rhythms are oscillations with a 24-h cycle that regulate various physiological processes (Gnocchi and Bruscalupi, 2017). These rhythms are driven by molecular clocks at the cellular level and regulated by a master clock located in the SCN of the hypothalamus (Spitschan and Joyce, 2023). The process by which light synchronizes these circadian rhythms is called photoentrainment (Duffy and Czeisler, 2009). This adaptation allows the body to align its internal clock with the external light-dark cycle (Astiz et al., 2019).

Human biology has evolved to follow these cycles to perform tasks optimally during certain times of the day (Finger and Kramer, 2021). The SCN, acting as a biological pacemaker, synchronizes the sleep-wake cycle and integrates environmental light inputs (Asgari-Targhi and Klerman, 2019; Gentry et al., 2021). The transmission of light signals to the SCN occurs via the retinohypothalamic tract (RHT), a pathway originating from the ipRGCs (Figure 1) (Díaz et al., 2016; Mure et al., 2019; Schoonderwoerd et al., 2022; Rabin et al., 2024).

ipRGCs play a central role in synchronizing circadian rhythms to environmental light. OPN4 is particularly sensitive to blue light (around 480 nm), which effectively suppresses melatonin release and shifts circadian phases (Roecklein et al., 2012). Variations in the OPN4 gene, such as P10L and I394T SNPs, may impact individuals’ sensitivity to light by altering the response properties of ipRGCs and making them more susceptible to mood disorders, sleep disturbances, or metabolic diseases (Rodgers et al., 2018a, 2018b). However, in modified mice, these polymorphisms did not significantly impair circadian photoentrainment or the pupillary light reflex, due to compensatory rods and cones. For instance, the pupillary light reflex has a fast component depending on cones and rods, and a slower component mediated by ipRGCs (Gooley et al., 2012; Lee et al., 2013). But, while the classical photoreceptors may compensate for some of the ipRGCs functions, for instance, the PPL as indicated above, the disruption in melanopsin signaling could still contribute to dysregulation of the circadian rhythm and specific diseases associated with SNPs because these variations on the melanopsin gene do alter the synaptic pathway of the RHT.

Exposure to light at specific times can shift the phases of circadian rhythms. For example, evening light exposure can delay sleep onset, while morning light helps advance the circadian clock, aligning it with daytime activities (Chellappa, 2021). However, disruptions to circadian rhythms through artificial light exposure or internal misalignments (e.g., due to sleep disorders) can negatively affect health by contributing to cardiovascular problems, cancer, obesity, type 2 diabetes (T2DM), and mood disorders through poor sleep quality (Foster, 2021).

OPN4 is critical in modulating circadian timing by regulating the body’s response to light. It modulates alertness, cognition, and the pupillary light reflex and helps synchronize physiological activities with the light-dark cycle (Rabin et al., 2024). By signaling light information to the SCN, OPN4 contributes to the adaptive timing of biological rhythms in response to environmental changes (Spitschan, 2019).

### Sleep, chronotype, and insomnia

An essential physiological process for mental and physical health is sleep. It influences hormone control, cognitive performance, immunological and cardiovascular health (Baranwal et al., 2023). Furthermore, it promotes neuroplasticity during the acquisition of new abilities or knowledge and is also involved in recovery roles in processes like hormone release and muscle repair (Vorster et al., 2024). However, since sleep and mental health are linked, an individual’s well-being may be compromised when they are affected by sleep disorders (Turkistani et al., 2023). In addition, sleeping disorders might raise the risk of developing conditions like anxiety, depression, bipolar disorders (Howarth and Miller, 2024), and even metabolic problems such as obesity, type 2 diabetes, and changes in intestinal microbiota (dos Santos and Galiè, 2024). Individual circadian rhythms and sleep-wake cycles can be affected by their chronotype, which is a particular preference for morning or evening activity (Montaruli et al., 2021). People with evening chronotypes tend to have major difficulty adjusting to society’s demands due to the earlier sleep-wake times, which makes them more prone to insomnia and sleep disruptions. This leads to negative health outcomes, including psychological distress (PD) and mood disorders (Bradford et al., 2021).

ipRGCs are highly involved in sleep regulation, and it has been shown in OPN4 knockout mice studies that affect REM and NREM sleep by mediating light-induced sleep (Wang et al., 2022) and evening light exposure can influence sleep parameters such as sleep efficiency and latency (Cajochen et al., 2022). Also, OPN4 direct light effects contribute to the sleep-wake cycle involved through circadian input, effects relayed by the SCN, subsequently suppressing melatonin (Hubbard et al., 2021).

Melatonin is a hormone secreted at night and has an important role in the processes regulating sleep and circadian rhythms (de Toledo et al., 2023). When exposed to blue light in the evening, sleep patterns are disrupted by the suppression of melatonin, affecting the circadian rhythm (Diaconu et al., 2023). Ziółkowska et al. (2022) showed that blue light exposure decreases OPN4 expression in the ipRGCs. Light absorption control can diminish the effects since it shortens sleep latency, decreases alertness, and reduces melatonin suppression (Schöllhorn et al., 2023).

According to Howarth and Miller (2024), mental health conditions can provoke a disruption in sleeping patterns, leading to shorter sleep duration. A comparison between the morning and evening chronotypes in the study of Ahn et al. (2024) showed that the morning type has better cognitive performance with less sleep (5–6 h), compared to the evening chronotype that required more hours of sleep to have a better cognitive function (7–8 h). Also, evening chronotypes display a decreased sleep quality, shorter sleep time, and overall sleep efficiency compared to the morning type (Colelli et al., 2023).

Sleep-wake patterns are complex and possess significant importance in different health conditions. It is characteristic of patients with epilepsy to exhibit disruptions in sleep-wake patterns, leading to reduced synchronization and a delay in the sleep phases (Liguori et al., 2022). It is also common in Alzheimer’s disease to have a sleep-wake disturbance that leads to memory loss. However, the specific patterns remain unclear (Falgàs et al., 2021). A hint can come from a study by La Morgia et al. (2023), who demonstrated that AD patients had a pronounced dysfunction of ipRGCs.

Parkinson’s disease is another neuropathy in which the involvement of ipRGCs has been demonstrated. It is known that patients suffer from sleep and circadian rhythm disorders, and in biopsies analyzed, there is a diminished amount or alteration in morphology of ipRGCs (La Morgia et al., 2023; Ortuño-Lizarán et al., 2018).

### Pupillary light reflex and ocular light perception disorders

Pupillary light reflex (PLR) is an involuntary response to changes in ambient light and is controlled by the autonomic nervous system (sympathetic and parasympathetic) (Hsu and Kuo, 2023). PLR has a complex role in the regulation of retinal illumination and the non-image forming (NIF) functions, and it has been found that PLR is useful to measure retinal irradiance to improve seasonal affective disorder analysis models (Klevens et al., 2024). The parameters by which PLR can be measured include initial pupil size, constriction velocity, dilation velocity, pupillary size, and ambient light conditions (Rosen et al., 2020). Some studies show that children at high risk of having an autism spectrum disorder (Kercher et al., 2020) and individuals with a history of concussions exhibit an altered PLR (Carrick et al., 2021). The role of ipRGCs in initiating this light response also has a plausible influence on the development of these dysfunctions.

Ambient light detection is possible due to photosensitive cells crucial for various physiological regulation processes. Cells that express melanopsin allow them to sense light directly (Lucas et al., 2020). And even though ipRGCs directly respond to light intensity for non-image forming functions, they can also receive input from cones and rods, contributing to the visual process (Mouland and Brown, 2022). Light detection is not limited to the retina since it has been shown that extraocular light perception can perform different functions in various tissues. Other opsins, particularly OPN3 and OPN5, have been shown to be involved in certain metabolic pathways (Andrabi et al., 2023). However, the mechanisms by which they work remain unknown.

## Discussion

This review investigated how polymorphisms in the OPN4 gene might influence or benefit the functioning of various physiological processes. The discovery and ongoing research on OPN4 have shown significant advances in photobiology. Many studies have supported the idea that this pigment was among the first ones distinguished from those found in cones and rods (Provencio and Warthen, 2012). Compared to other opsins primarily involved in image formation, OPN4 containing cells function more as photoreceptors for non-visual tasks, including the previously mentioned ones: photoentrainment, PLR, and potential effects from light sensitivity, such as SAD, insomnia, and even associations with metabolism, obesity, and type 2 diabetes (Fleury et al., 2020).

The discovery of OPN4 opened various new areas of research that can be further developed in chronobiology, neuroscience, and even medicine. Studies have supported its role in regulating circadian rhythms due to its direct connection with the SCN and its implications in sleep disorders and related health issues (Feigl et al., 2020). Despite this, specific mechanisms remain to be fully elucidated. On the other hand, research has also explored the effects of blue light on the sleep-wake cycle and how OPN4 may help mitigate sleep issues (Diaconu et al., 2023). OPN4 is not only found in the retina, but it has also drawn attention to its possible implications in the skin and adipose tissue. However, to better understand the full impact, it is necessary to continue developing models of the potential mechanisms through which OPN4 functions in various regions (Kankanamge et al., 2018; Pan et al., 2023). These results suggest the opsin’s possible susceptibility to gene changes due to one or more SNPs.

The review identified several SNPs with associations related to sleep and the PLR (Table 2). Overall, we found mixed results for efficacy and comparison studies, as a few of these variations already have evidence of their potential implications for human function and health, while others remain unknown (Table 1). Nonetheless, no mechanisms have been definitively established to explain how these mutations in the gene might cause functional changes. Accordingly, Rodgers et al. (2018a, 2018b) showed an association between light sensitivity and altered OPN4 signaling, suggesting that protein function can be affected by the OPN4 SNPs. This might suggest that changes in specific parts of the phototransduction process could disrupt its ability to maintain consistent photoentrainment or even modify the cellular response to light. This modification can potentially lead to a decrease, increase, overexpression, or suppression of certain hormones, enzymes, or proteins.

**Table 2:** Single-nucleotide polymorphism associations.

Studies	SNPs	Associations
Roecklein et al. (2009) Lee et al. (2013) Higuchi et al. (2013) Rodgers et al. (2018a) Rodgers et al. (2018b) Gutiérrez-Amavizca et al. (2021)	P10L (rs2675703)	Seasonal affective disorder (SAD), chronotype and chronic insomnia
Smieszek et al. (2022)	R168C (rs143641898)	Delayed sleep-wake phase disorder (DSWPD)
Rodgers et al. (2018b)	L365V	Not reported
Roecklein et al. (2009) Lee et al. (2013) Higuchi et al. (2013) Lee et al. (2014) Rodgers et al. (2018a) Rodgers et al. (2018b)	I394T (rs1079610)	Pupillary light response (PLR) and sleep/wake timing
Rodgers et al. (2018b)	R406W	Not reported
Roecklein et al. (2009) Rodgers et al. (2018b)	D444G (rs12262894)	Not reported

Regarding the therapeutic perspective, the functions and associations of OPN4, along with the alterations caused by its SNPs, are valuable due to their connections to health issues such as Alzheimer’s disease, Parkinson’s disease (La Morgia et al., 2017, 2023) and autism spectrum disorder (Kercher et al., 2020). Results also show potential links to increased body weight that could lead to obesity or type 2 diabetes. In the study of Gutiérrez-Amavizca et al. (2021), it was observed that individuals with chronic insomnia associated with the P10L SNP predominantly belonged to a population with these problems. Hence, there is a strong interest in further exploring these associations to develop targeted therapeutic interventions. Photobiomodulation (PBM) is one of the therapies based on light that can potentially help treat these conditions. The suggested role of the therapy in restoring homeostasis must occur because it has opsins and light-sensitive organelles, acting by different wavelengths, duration, and intensity (Galache et al., 2024; Santana-Blank and Rodríguez-Santana, 2018). However, it is still under development, and there is a need to use standardized protocols as a therapy for these conditions (Conus and Geiser, 2020).

## Conclusion

This systematic review aimed to gather available information on the OPN4 gene SNPs and their impact on the health of carriers. Our review revealed that P10L, I394T, and R168C variants are associated with affective states, changes in chronotype, and sleep disorders. While some SNPs in the OPN4 gene have well-known associations, like those studied here, others remain unknown or have unclear effects on the gene, as most of those already reported. Additionally, we did not find any reports studying the function and association of OPN4 located in different body regions. To address these limitations, future research should focus on assessing more SNPs outside the eye, with associations that have not been previously tested, but that OPN4 has.

In summary, while the current review highlights the available information and possible applications, the field of this gene and its variations is still wide open since no clear understanding of the functioning of OPN4 containing these SNPs has been established.

## References

[ref1] Ahn E. K. Yoon K. Park J.-E. (2024). Association between sleep hours and changes in cognitive function according to the morningness-eveningness type: a population-based study. J. Affect. Disord. 345, 112–119. doi: 10.1016/j.jad.2023.10.122, PMID: 37865346

[ref2] Andrabi M. Upton B. A. Lang R. A. Vemaraju S. (2023). An expanding role for nonvisual opsins in extraocular light sensing physiology. Annu. Rev. Vis. Sci. 9, 245–267. doi: 10.1146/annurev-vision-100820-094018 37196422

[ref3] Asgari-Targhi A. Klerman E. B. (2019). Mathematical modeling of circadian rhythms. Wiley Interdiscip. Rev. Syst. Biol. Med. 11:e1439. doi: 10.1002/wsbm.1439, PMID: 30328684 PMC6375788

[ref4] Astiz M. Heyde I. Oster H. (2019). Mechanisms of communication in the mammalian circadian timing system. Int. J. Mol. Sci. 20:343. doi: 10.3390/ijms20020343, PMID: 30650649 PMC6359556

[ref5] Baranwal N. Yu P. K. Siegel N. S. (2023). Sleep physiology, pathophysiology, and sleep hygiene. Prog. Cardiovasc. Dis. 77, 59–69. doi: 10.1016/j.pcad.2023.02.005, PMID: 36841492

[ref6] Bellingham J. Chaurasia S. S. Melyan Z. Liu C. Cameron M. A. Tarttelin E. E. . (2006). Evolution of melanopsin photoreceptors: discovery and characterization of a new melanopsin in nonmammalian vertebrates. PLoS Biol. 4:e254. doi: 10.1371/journal.pbio.0040254, PMID: 16856781 PMC1514791

[ref7] Bidaki R. Zarei M. Khorram Toosi A. Hakim Shooshtari M. (2012). A review on genetics of sleep disorders. Iran. J. Psychiatry Behav. Sci. 6, 12–19. 24644464 PMC3939950

[ref8] Bradford D. R. R. Biello S. M. Russell K. (2021). Insomnia symptoms mediate the association between eveningness and suicidal ideation, defeat, entrapment, and psychological distress in students. Chronobiol. Int. 38, 1397–1408. doi: 10.1080/07420528.2021.1931274, PMID: 34100311

[ref9] Buhr E. D. (2023). Tangled up in blue: contribution of short-wavelength sensitive cones in human circadian photoentrainment. Proc. Natl. Acad. Sci. U.S.A. 120:e2219617120. doi: 10.1073/pnas.2219617120, PMID: 36598954 PMC9926240

[ref10] Cajochen C. Stefani O. Schöllhorn I. Lang D. Chellappa S. (2022). Influence of evening light exposure on polysomnographically assessed night-time sleep: a systematic review with meta-analysis. Light. Res. Technol. 54, 609–624. doi: 10.1177/14771535221078765

[ref11] Carrick F. R. Azzolino S. F. Hunfalvay M. Pagnacco G. Oggero E. D’Arcy R. C. N. . (2021). The pupillary light reflex as a biomarker of concussion. Life 11:1104. doi: 10.3390/life11101104, PMID: 34685475 PMC8537991

[ref12] Chellappa S. L. (2021). Individual differences in light sensitivity affect sleep and circadian rhythms. Sleep 44:zsaa214. doi: 10.1093/sleep/zsaa214, PMID: 33049062 PMC7879412

[ref13] Colelli D. R. Dela Cruz G. R. Kendzerska T. Murray B. J. Boulos M. I. (2023). Impact of sleep chronotype on in-laboratory polysomnography parameters. J. Sleep Res. 32:e13922. doi: 10.1111/jsr.13922, PMID: 37150591

[ref14] Conus V. Geiser M. (2020). A review of silent substitution devices for melanopsin stimulation in humans. Photonics 7:121. doi: 10.3390/photonics7040121

[ref15] de Toledo L. H. S. Moraes M. N. Poletini M. d. O. Neto J. C. Baron J. Mota T. (2023). Modeling the influence of nighttime light on melatonin suppression in humans: milestones and perspectives. J. Photochem. Photobiol. 16:100199. doi: 10.1016/j.jpap.2023.100199

[ref16] Diaconu G.-A. Iordăchel C. M. Coca C. Feraru N. Gheorghevici C. Zisu D. . (2023). Blue light and its effects on sleep. Pneumologia 72, 39–43. doi: 10.2478/pneum-2024-0006

[ref17] dos Santos A. Galiè S. (2024). The microbiota-gut-brain axis in metabolic syndrome and sleep disorders: a systematic review. Nutrients 16:390. doi: 10.3390/nu16030390, PMID: 38337675 PMC10857497

[ref18] Duffy J. F. Czeisler C. A. (2009). Effect of light on human circadian physiology. Sleep Med. Clin. 4, 165–177. doi: 10.1016/j.jsmc.2009.01.004, PMID: 20161220 PMC2717723

[ref19] Falgàs N. Walsh C. M. Neylan T. C. Grinberg L. T. (2021). Deepen into sleep and wake patterns across Alzheimer’s disease phenotypes. Alzheimers Dement. 17, 1403–1406. doi: 10.1002/alz.12304, PMID: 33710762 PMC8364869

[ref20] Feigl B. Dumpala S. Kerr G. K. Zele A. J. (2020). Melanopsin cell dysfunction is involved in sleep disruption in Parkinson’s disease. J. Parkinsons Dis. 10, 1467–1476. doi: 10.3233/JPD-202178, PMID: 32986681

[ref21] Finger A. M. Kramer A. (2021). Mammalian circadian systems: organization and modern life challenges. Acta Physiol. 231:e13548. doi: 10.1111/apha.13548, PMID: 32846050

[ref22] Fleury G. Masís-Vargas A. Kalsbeek A. (2020). Metabolic implications of exposure to light at night: lessons from animal and human studies. Obesity 28, S18–S28. doi: 10.1002/oby.22807, PMID: 32700826 PMC7497102

[ref23] Foster R. G. (2021). Fundamentals of circadian entrainment by light. Light. Res. Technol. 53, 377–393. doi: 10.1177/14771535211014792

[ref24] Foster R. G. Hughes S. Peirson S. N. (2020). Circadian photoentrainment in mice and humans. Biology 9:180. doi: 10.3390/biology9070180, PMID: 32708259 PMC7408241

[ref25] Gagné A.-M. Lévesque F. Gagné P. Hébert M. (2011). Impact of blue vs. red light on retinal response of patients with seasonal affective disorder and healthy controls. Prog. Neuropsychopharmacol. Biol. Psychiatry 35, 227–231. doi: 10.1016/j.pnpbp.2010.11.009, PMID: 21094670

[ref26] Galache T. R. Sena M. M. Tassinary J. A. F. Pavani C. (2024). Photobiomodulation for melasma treatment: integrative review and state of the art. Photodermatol. Photoimmunol. Photomed. 40:e12935. doi: 10.1111/phpp.12935, PMID: 38018017

[ref27] Gentry N. W. Ashbrook L. H. Fu Y. H. Ptáček L. J. (2021). Human circadian variations. J. Clin. Invest. 131:e148282. doi: 10.1172/JCI148282, PMID: 34396981 PMC8363277

[ref28] Gnocchi D. Bruscalupi G. (2017). Circadian rhythms and hormonal homeostasis: pathophysiological implications. Biology 6:10. doi: 10.3390/biology6010010, PMID: 28165421 PMC5372003

[ref29] Gooley J. J. Ho Mien I. St. Hilaire M. A. Yeo S.-C. Chua E. C.-P. van Reen E. . (2012). Melanopsin and rod-cone photoreceptors play different roles in mediating pupillary light responses during exposure to continuous light in humans. J. Neurosci. 32, 14242–14253. doi: 10.1523/JNEUROSCI.1321-12.2012, PMID: 23055493 PMC3515688

[ref30] Gutiérrez-Amavizca B. E. de Oca E. P. M. Gutiérrez-Amavizca J. P. Castro O. D. Ruíz-Marquez C. H. Conde-Andreu K. P. . (2021). Association of P10l polymorphism in melanopsin gene with chronic insomnia in Mexicans. Int. J. Environ. Res. Public Health 18:571. doi: 10.3390/ijerph18020571 33445464 PMC7827055

[ref31] Haltaufderhyde K. Ozdeslik R. N. Wicks N. L. Najera J. A. Oancea E. (2015). Opsin expression in human epidermal skin. Photochem. Photobiol. 91, 117–123. doi: 10.1111/php.12354, PMID: 25267311 PMC4303996

[ref32] Higuchi S. Hida A. Tsujimura S. I. Mishima K. Yasukouchi A. Lee S. I. . (2013). Melanopsin gene polymorphism I394T is associated with pupillary light responses in a dose-dependent manner. PLoS One 8:e60310. doi: 10.1371/journal.pone.0060310, PMID: 23555953 PMC3610661

[ref33] Howarth N. E. Miller M. A. (2024). Sleep, sleep disorders, and mental health: a narrative review. Heart Mind 8, 146–158. doi: 10.4103/hm.HM-D-24-00030

[ref34] Hsu C.-H. Kuo L.-T. (2023). Application of Pupillometry in Neurocritical patients. J. Pers. Med. 13:1100. doi: 10.3390/jpm13071100, PMID: 37511713 PMC10381796

[ref35] Hubbard J. Kobayashi Frisk M. Ruppert E. Tsai J. W. Fuchs F. Robin-Choteau L. . (2021). Dissecting and modeling photic and melanopsin effects to predict sleep disturbances induced by irregular light exposure in mice. Proc. Natl. Acad. Sci. US.A. 118:e2017364118. doi: 10.1073/pnas.2017364118, PMID: 34155139 PMC8237663

[ref36] Kankanamge D. Ratnayake K. Samaradivakara S. Karunarathne A. (2018). Melanopsin (Opn4) utilizes Gαi and gβγ as major signal transducers. J. Cell Sci. 131:jcs212910. doi: 10.1242/jcs.212910, PMID: 29712722

[ref37] Karki R. Pandya D. Elston R. C. Ferlini C. (2015). Defining “mutation” and “polymorphism” in the era of personal genomics. BMC Med. Genet. 8:37. doi: 10.1186/s12920-015-0115-z, PMID: 26173390 PMC4502642

[ref38] Karthikeyan R. Davies W. I. L. Gunhaga L. (2023). Non-image-forming functional roles of OPN3, OPN4 and OPN5 photopigments. J. Photochem. Photobiol. 15:100177. doi: 10.1016/j.jpap.2023.100177

[ref39] Kercher C. Azinfar L. Dinalankara D. M. R. Takahashi T. N. Miles J. H. Yao G. (2020). A longitudinal study of pupillary light reflex in 6- to 24-month children. Sci. Rep. 10:1205. doi: 10.1038/s41598-020-58254-6, PMID: 31988320 PMC6985190

[ref40] Klevens A. M. Taylor M. L. Wescott D. L. Gamlin P. D. Franzen P. L. Hasler B. P. . (2024). The role of retinal irradiance estimates in melanopsin-driven retinal responsivity: a reanalysis of the post-illumination pupil response in seasonal affective disorder. Sleep 47:zsae109. doi: 10.1093/sleep/zsae109, PMID: 38877879 PMC11381569

[ref41] Kusumoto J. Takeo M. Hashikawa K. Komori T. Tsuji T. Terashi H. . (2020). OPN4 belongs to the photosensitive system of the human skin. Genes Cells 25, 215–225. doi: 10.1111/gtc.12751, PMID: 31989708

[ref42] La Morgia C. Mitolo M. Romagnoli M. Stanzani Maserati M. Evangelisti S. De Matteis M. . (2023). Multimodal investigation of melanopsin retinal ganglion cells in Alzheimer’s disease. Ann. Clin. Transl. Neurol. 10, 918–932. doi: 10.1002/acn3.51773 37088544 PMC10270274

[ref43] La Morgia C. Ross-Cisneros F. N. Sadun A. A. Carelli V. (2017). Retinal ganglion cells and circadian rhythms in Alzheimer’s disease, Parkinson’s disease, and beyond. Front. Neurol. 8, –162. doi: 10.3389/fneur.2017.00162, PMID: 28522986 PMC5415575

[ref44] Lee S. Hida A. Kitamura S. Mishima K. Higuchi S. (2014). Association between the melanopsin gene polymorphism OPN4*Ile394Thr and sleep/wake timing in Japanese university students. J. Physiol. Anthropol. 33:9. doi: 10.1186/1880-6805-33-9, PMID: 24887407 PMC4048048

[ref45] Lee S. I. Hida A. Tsujimura S. Morita T. Mishima K. Higuchi S. (2013). Association between melanopsin gene polymorphism (I394T) and pupillary light reflex is dependent on light wavelength. J. Physiol. Anthropol. 32:16. doi: 10.1186/1880-6805-32-16, PMID: 24119231 PMC4015917

[ref46] Leung N. Y. Montell C. (2017). Unconventional roles of opsins. Annu. Rev. Cell Dev. Biol. 33, 241–264. doi: 10.1146/annurev-cellbio-100616-060432 28598695 PMC5963513

[ref47] Liguori C. Spanetta M. Fernandes M. Izzi F. Placidi F. Mercuri N. B. (2022). More than sleep and wake disturbances: an actigraphic study showing the sleep-wake pattern dysregulation in epilepsy. Seizure 94, 95–99. doi: 10.1016/j.seizure.2021.11.024, PMID: 34883462

[ref48] Lucas R. J. Allen A. E. Milosavljevic N. Storchi R. Woelders T. (2020). Can we see with melanopsin? Annu. Rev. Vis. Sci. 6, 453–468. doi: 10.1146/annurev-vision-030320-041239, PMID: 32491960

[ref49] Lucas R. J. Hattar S. Takao M. Berson D. M. Foster R. G. Yau K. W. (2003). Diminished pupillary light reflex at high irradiances in melanopsin-knockout mice. Science 299, 245–247. doi: 10.1126/science.1077293, PMID: 12522249

[ref50] McGuinness L. A. Higgins J. P. T. (2021). Risk-of-bias VISualization (robvis): an R package and shiny web app for visualizing risk-of-bias assessments. Res. Synth. Methods 12, 55–61. doi: 10.1002/jrsm.1411, PMID: 32336025

[ref51] Montaruli A. Castelli L. Mulè A. Scurati R. Esposito F. Galasso L. . (2021). Biological rhythm and chronotype: new perspectives in health. Biomol. Ther. 11:487. doi: 10.3390/biom11040487, PMID: 33804974 PMC8063933

[ref52] Moraes M. N. de Assis L. V. M. Provencio I. Castrucci A. M. L. (2021). Opsins outside the eye and the skin: a more complex scenario than originally thought for a classical light sensor. Cell Tissue Res. 385, 519–538. doi: 10.1007/s00441-021-03500-0, PMID: 34236517

[ref53] Mouland J. W. Brown T. M. (2022). Beyond irradiance: visual signals influencing mammalian circadian function. Prog. Brain Res. 273, 145–169. doi: 10.1016/bs.pbr.2022.04.010 35940714

[ref54] Nissilä J. S. Mänttäri S. K. Särkioja T. T. Tuominen H. J. Takala T. E. Kiviniemi V. J. . (2017). The distribution of melanopsin (OPN4) protein in the human brain. Chronobiol. Int. 34, 37–44. doi: 10.1080/07420528.2016.1232269, PMID: 27690288

[ref55] Ondrusova K. Fatehi M. Barr A. Czarnecka Z. Long W. Suzuki K. . (2017). Subcutaneous white adipocytes express a light sensitive signaling pathway mediated via a melanopsin/TRPC channel axis. Sci. Rep. 7:16332. doi: 10.1038/s41598-017-16689-4, PMID: 29180820 PMC5703708

[ref56] Ortuño-Lizarán I. Esquiva G. Beach T. G. Serrano G. E. Adler C. H. Lax P. . (2018). Degeneration of human photosensitive retinal ganglion cells may explain sleep and circadian rhythms disorders in Parkinson’s disease. Acta Neuropathol. Commun. 6:90. doi: 10.1186/s40478-018-0596-z, PMID: 30201049 PMC6130068

[ref57] Page M. J. McKenzie J. E. Bossuyt P. M. Boutron I. Hoffmann T. C. Mulrow C. D. . (2021). The PRISMA 2020 statement: an updated guideline for reporting systematic reviews. PLoS Med. 18:e1003583. doi: 10.1371/journal.pmed.1003583, PMID: 33780438 PMC8007028

[ref58] Pan D. Wang Z. Chen Y. Cao J. (2023). Melanopsin-mediated optical entrainment regulates circadian rhythms in vertebrates. Commun. Biol. 6:1054. doi: 10.1038/s42003-023-05432-7, PMID: 37853054 PMC10584931

[ref59] Provencio I. Jiang G. De Grip W. J. Hayes W. P. Rollag M. D. (1998). Melanopsin: an opsin in melanophores, brain, and eye. Proc. Natl. Acad. Sci. 95, 340–345. doi: 10.1073/pnas.95.1.340, PMID: 9419377 PMC18217

[ref60] Provencio I. Warthen D. M. (2012). Melanopsin, the photopigment of intrinsically photosensitive retinal ganglion cells. Wiley Interdiscip. Rev. Membr. Transp. Signaling 1, 228–237. doi: 10.1002/wmts.29, PMID: 40432551

[ref61] Rabin J. Lundby J. Brase M. Vu A. Millis A. Wood G. . (2024). Vision within the blind spot: a new test to quantify melanopsin pathway sensitivity. Eye 38, 2653–2655. doi: 10.1038/s41433-024-03102-4, PMID: 38698051 PMC11383934

[ref62] Rodgers J. Hughes S. Pothecary C. A. Brown L. A. Hickey D. G. Peirson S. N. . (2018a). Defining the impact of melanopsin missense polymorphisms using *in vivo* functional rescue. Hum. Mol. Genet. 27, 2589–2603. doi: 10.1093/hmg/ddy150, PMID: 29718372 PMC6048994

[ref63] Rodgers J. Peirson S. N. Hughes S. Hankins M. W. (2018b). Functional characterisation of naturally occurring mutations in human melanopsin. Cell. Mol. Life Sci. 75, 3609–3624. doi: 10.1007/s00018-018-2813-0, PMID: 29700553 PMC6133154

[ref64] Roecklein K. A. Rohan K. J. Duncan W. C. Rollag M. D. Rosenthal N. E. Lipsky R. H. . (2009). A missense variant (P10L) of the melanopsin (OPN4) gene in seasonal affective disorder. J. Affect. Disord. 114, 279–285. doi: 10.1016/j.jad.2008.08.005, PMID: 18804284 PMC2647333

[ref65] Roecklein K. Wong P. Ernecoff N. Miller M. Donofry S. Kamarck M. . (2013). The post illumination pupil response is reduced in seasonal affective disorder. Psychiatry Res. 210, 150–158. doi: 10.1016/j.psychres.2013.05.023, PMID: 23809464 PMC3795919

[ref66] Roecklein K. A. Wong P. M. Franzen P. L. Hasler B. P. Wood-Vasey W. M. Nimgaonkar V. L. . (2012). Melanopsin gene variations interact with season to predict sleep onset and chronotype. Chronobiol. Int. 29, 1036–1047. doi: 10.3109/07420528.2012.706766, PMID: 22881342 PMC3724237

[ref67] Rosen J. Privitera C. Bulmus R. Nakamura M. Hartmann A. (2020). The photomotor response—dynamic quantification by a portable pupillometer. Am. J. Intern. Med. 8:230. doi: 10.11648/j.ajim.20200805.17

[ref68] Santana-Blank L. Rodríguez-Santana E. (2018). Photobiomodulation in light of our biological clock’s inner workings. Photomed. Laser Surg. 36, 119–121. doi: 10.1089/pho.2018.4445, PMID: 29649380 PMC5863078

[ref69] Schöllhorn I. Stefani O. Lucas R. J. Spitschan M. Slawik H. C. Cajochen C. (2023). Melanopic irradiance defines the impact of evening display light on sleep latency, melatonin and alertness. Commun. Biol. 6:228. doi: 10.1038/s42003-023-04598-4, PMID: 36854795 PMC9974389

[ref70] Schoonderwoerd R. A. de Rover M. M. Janse J. A. Hirschler L. Willemse C. R. Scholten L. . (2022). The photobiology of the human circadian clock. Proc. Natl. Acad. Sci U.S.A. 119:e2118803119. doi: 10.1073/pnas.2118803119 35312355 PMC9060497

[ref71] Smieszek S. P. Polymeropoulos C. M. Birznieks G. Polymeropoulos M. H. (2022). Case report: A novel missense variant in melanopsin associates with delayed sleep phenotype: whole genome sequencing study. Front. Genet. 13:896192. doi: 10.3389/fgene.2022.896192, PMID: 36246649 PMC9561615

[ref72] Spitschan M. (2019). Melanopsin contributions to non-visual and visual function. Curr. Opin. Behav. Sci. 30, 67–72. doi: 10.1016/j.cobeha.2019.06.004, PMID: 31396546 PMC6687502

[ref73] Spitschan M. Joyce D. S. (2023). Human-centric lighting research and policy in the melanopsin age. Policy Insights Behav. Brain Sci. 10, 237–246. doi: 10.1177/23727322231196896, PMID: 38919981 PMC7615961

[ref74] Turkistani O. Albalawi A. Thabit R. Alamri H. Alshehri N. Alsufyani S. . (2023). Relationship between sleep disorders and mental health. J. Healthcare Sci. 3, 163–166. doi: 10.52533/JOHS.2023.30601

[ref75] Vorster A. P. A. van Someren E. J. W. Pack A. I. Huber R. Schmidt M. H. Bassetti C. L. A. (2024). Sleep health. Clin. Transl. Neurosci. 8:8. doi: 10.3390/ctn8010008

[ref76] Wang Y. Yang W. Zhang P. Ding Z. Wang L. Cheng J. (2022). Effects of light on the sleep-wakefulness cycle of mice mediated by intrinsically photosensitive retinal ganglion cells. Biochem. Biophys. Res. Commun. 592, 93–98. doi: 10.1016/j.bbrc.2022.01.023, PMID: 35033872

[ref77] Ziółkowska N. Chmielewska-Krzesińska M. Vyniarska A. Sienkiewicz W. (2022). Exposure to blue light reduces melanopsin expression in intrinsically photoreceptive retinal ganglion cells and damages the inner retina in rats. Invest. Ophthalmol. Vis. Sci. 63:26. doi: 10.1167/iovs.63.1.26, PMID: 35060997 PMC8787613

